# Biological Effects on S-PRG: An Integrative Review

**DOI:** 10.3390/jfb17040182

**Published:** 2026-04-09

**Authors:** Hudson Balthazar Cavalcante de Oliveira, Jessica Zablocki da Luz, Fabio Eduardo de Lima, Cauani de Castro Busatto Fernandes, Leticia Barbosa Wetter, Carolina Silva Schiebel, André Vieira Souza, Fhernanda Ribeiro Smiderle, Daniele Maria-Ferreira, Cleber Machado-Souza

**Affiliations:** 1 Hospital Pequeno Príncipe, Rua Desembargador Motta, 1070, Água Verde, Curitiba 80250-060, PR, Brazil; hudson.oliveira@hpp.org.br (H.B.C.d.O.);; 2 Instituto de Pesquisa Pelé Pequeno Príncipe, Av. Silva Jardim, 1632, Água Verde, Curitiba 80250-060, PR, Brazil; 3 Faculdades Pequeno Príncipe, Av. Iguaçu, 333, Rebouças, Curitiba 80230-020, PR, Brazil

**Keywords:** biomaterial, biological activity, S-PRG

## Abstract

Advances in dental material science over recent decades have significantly improved the mechanical, physical, esthetic, and adhesive properties of restorative systems. As clinical performance and durability have reached high standards, research has progressively shifted from purely mechanical replacement toward the development of bioactive materials capable of interacting beneficially with biological tissues. Rather than functioning solely as passive restoratives, contemporary materials are increasingly designed to contribute to disease prevention and tissue repair. Bioactive functionality encompasses both bioprotective and biopromotive effects, including antimicrobial activity, reinforcement of the dental substrate, promotion of remineralization, modulation of inflammatory responses, and stimulation of regenerative pathways. In this context, the surface pre-reacted glass ionomer (S-PRG) particle has emerged as a multifunctional bioactive technology. Its unique three-layer structure enables sustained release of multiple ions, fluoride, strontium, boron, sodium, silicate, and aluminum, associated with mineralization, biofilm inhibition, inflammatory regulation, and activation of cellular signaling pathways. An integrative review was conducted through a literature search in PubMed, SciELO and Scopus using the descriptors “Surface-reaction-type prereacted glass ionomer” and “S-PRG.” Experimental studies evaluating antimicrobial, anti-inflammatory, remineralizing, cellular, or regenerative effects of S-PRG-containing materials were considered eligible. A total of 49 studies met the inclusion criteria and were analyzed through descriptive synthesis. The available evidence indicates that the biological activity of S-PRG-containing materials extends beyond caries prevention, including modulation of inflammatory responses, enhancement of mineralization processes, and stimulation of cellular pathways related to tissue repair. These findings highlight the potential of S-PRG technology as a promising strategy for the development of restorative materials with regenerative and preventive properties.

## 1. Introduction

Over the past decades, technological advances have substantially improved the mechanical, physical, esthetic, and adhesive properties of dental materials. Contemporary restorative systems exhibit excellent clinical performance and durability, which has progressively shifted the focus of material development beyond mechanical replacement toward bioactive functionality [1]. Rather than serving solely as passive restorative substitutes, modern dental materials are increasingly expected to interact beneficially with biological tissues, contributing to disease prevention and tissue repair.

Bioactive functionality encompasses both bioprotective and biopromotive effects. Bioprotective actions include the control of bacterial infection and reinforcement of the dental substrate [2], whereas biopromotive effects involve remineralization [3], modulation of inflammatory responses [4,5], and activation of cellular pathways associated with tissue repair [6,7]. These biological interactions reflect a broader paradigm shift in restorative dentistry, from extensive and aggressive tissue removal toward minimally invasive and conservative approaches aimed at preserving healthy dental structure.

This transition aligns with the growing emphasis on patient-oriented care and personalized oral health strategies. Both self-care and professional interventions increasingly incorporate bioactive technologies as adjunctive tools in preventive and therapeutic protocols [8,9,10,11,12,13]. However, despite the widespread use of the term “bioactive,” its definition in dentistry remains under debate. In a recent review, Imazato, Nakatsuka [13] highlighted the lack of a standardized definition within the dental field. Outside dentistry, bioactivity generally refers to materials capable of eliciting positive biological responses through interactions with cells and tissues.

To address this conceptual gap, the FDI, World Dental Federation, published a consensus statement outlining essential criteria for bioactive restorative materials [1]. According to this framework, bioactivity in dentistry should be supported by biological, chemical, or combined mechanisms and aligned with four principal therapeutic objectives: (1) promotion of mineralization or hard tissue formation; (2) control of bacterial infection; (3) prevention or modulation of inflammation; and (4) promotion of tissue regeneration.

Within this context, the surface pre-reacted glass ionomer (S-PRG) particle has emerged as a multifunctional bioactive technology. Through its sustained multi-ion release system, comprising fluoride, strontium, boron, sodium, aluminum, and silicon, S-PRG exhibits a broad spectrum of biological effects, including acid neutralization, enamel remineralization, antimicrobial and antifungal activity, modulation of inflammatory pathways, enhancement of epithelial barrier function, and activation of cellular signaling mechanisms associated with migration and differentiation.

Considering these diverse biological interactions, it is plausible to hypothesize that the S-PRG particle may extend beyond preventive applications and play a significant role in tissue regeneration through modulation of gene expression, intracellular signaling pathways, and controlled inflammatory responses. However, most of the available evidence derives from experimental studies, and the clinical relevance of these mechanisms remains to be established.

Thus, this integrative review aims to synthesize the current scientific evidence regarding the biological activities associated with the S-PRG particle (Shofu Inc., Kyoto, Japan), integrating findings from experimental and translational studies to support its potential applicability in clinical practice, particularly with respect to its regenerative properties.

## 2. Materials and Methods

An integrative review was conducted to synthesize the available scientific evidence regarding the biological activities associated with the surface pre-reacted glass ionomer (S-PRG) particle. Searches were performed in PubMed, SciELO and Scopus using the descriptors “Surface-reaction-type prereacted glass ionomer” and “S-PRG”, covering studies published between 2012 and 2026. Only peer-reviewed papers were considered; therefore, gray literature, conference proceedings, and manufacturer-sponsored reports were not included. Eligible studies included experimental investigations evaluating antimicrobial, anti-inflammatory, remineralizing, cellular, or regenerative effects of S-PRG-containing materials. These comprised in vitro studies, in vivo animal studies, in situ human biofilm models, and clinical investigations, primarily within dental applications. Review papers were not included, as the focus was on primary experimental evidence.

As this study was designed as an unstructured integrative review with a descriptive synthesis of the literature, the methodology did not follow systematic review reporting frameworks such as PRISMA, and therefore procedures typical of systematic reviews (e.g., PRISMA flow diagram, formal risk-of-bias assessment, duplicate screening, or inter-reviewer agreement analysis) were not applied. The hierarchy of evidence was adopted as a descriptive framework to organize and compare the included studies according to their methodological design. The levels were defined as follows: Level 1: meta-analyses of multiple randomized controlled clinical trials; Level 2: individual experimental studies; Level 3: quasi-experimental studies; Level 4: descriptive (non-experimental) or qualitative studies; Level 5: case or experience reports; and Level 6: expert opinion.

Each included study was categorized according to this hierarchy based on its methodological design for descriptive comparison across studies.

Data extraction and synthesis were performed descriptively, focusing on biological mechanisms, experimental models, material formulations, and reported outcomes.

## 3. Results

### 3.1. Selected Studies

Forty-nine articles investigating the association between S-PRG and biological activity were included in this study, identified through searches performed in PubMed, SciELO, and Scopus using the descriptors “Surface-reaction-type prereacted glass ionomer” and “S-PRG.” Although the inclusion of additional databases and expanded search strategies could further enhance the comprehensiveness of the evidence retrieval, the selected databases provide broad coverage of the relevant dental and biomaterials literature. Among the included studies, three were published in 2017, seven in 2018, six in 2019, four in 2020, two in 2021, five in 2022, two in 2023, six in 2024, four in 2025, and two in 2026. Additionally, earlier contributions included two studies from 2012, one from 2014, one from 2015, and four from 2016. This distribution demonstrates a clear increase in scientific interest over the past decade, particularly from 2018 onward, reflecting the growing expansion of research on the biological properties and clinical potential of S-PRG technology.

After applying the eligibility criteria and analyzing the methodological design of the included papers, all selected studies were classified as Level 2, as they consisted of individual experimental investigations. No meta-analyses, randomized controlled clinical trials, quasi-experimental studies, descriptive studies, or expert-opinion reports specifically addressing the biological mechanisms of S-PRG particles were identified within the scope of this review.

To provide greater analytical clarity and translational perspective, the Level 2 studies were further organized into methodological subcategories according to experimental design: (i) in vitro studies (cell culture, biofilm, and mechanistic assays); (ii) in vivo animal studies; (iii) in situ human biofilm models; and (iv) clinical investigations. These subcategories do not represent different strengths within the formal hierarchy but were adopted to facilitate the interpretation of translational progression from laboratory findings to clinical application. Table 1 presents the studies included in this review.

### 3.2. The S-PRG Particle

One strategy for developing bioactive dental materials involves incorporating particles capable of releasing biologically active components. Several approaches have been described in the literature for designing functional fillers, with particular emphasis on inorganic particles engineered to release specific therapeutic ions in a controlled manner. Among these technologies, the surface pre-reacted glass ionomer (S-PRG) particle, developed by Shofu Inc., was introduced to the market in 2000 through resin composite materials incorporating this bioactive system.

**Figure 1:**
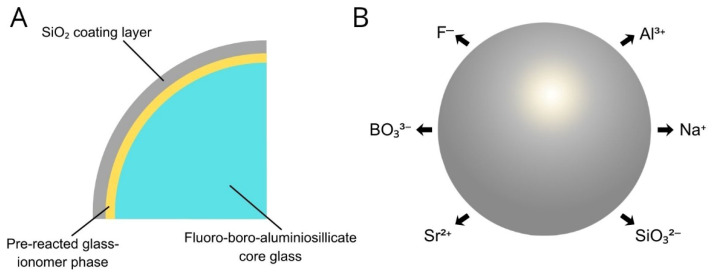
Surface Pre-Reacted Glass Ionomer (S-PRG) Filler. (**A**) The S-PRG filler exhibits a trilaminar structure consisting of an outer silica (SiO_2_) coating layer, an intermediate pre-reacted glass ionomer phase, and an inner functional fluoro-boro-aluminosilicate glass core. (**B**) This structure enables the sustained release of multiple bioactive ions, including strontium (Sr^2+^), borate (BO_3_^3−^), fluoride (F^−^), sodium (Na^+^), silicate (SiO_3_^2−^), and aluminum (Al^3+^). Adapted from Shofu Inc.

The S-PRG particle is characterized by a unique three-layer structure (Figure 1A). The outermost layer consists of a silica (SiO_2_) coating, beneath which lies a pre-reacted glass ionomer phase surrounding a fluoro-boro-aluminosilicate glass core. The pre-reacted layer is formed by spraying polyacrylic acid onto the glass particles, allowing the acid to penetrate the silica coating and initiate an acid-base reaction with the glass core. This controlled pre-reaction creates a stable glass ionomer phase at the particle surface while preserving the structural integrity of the core.

This architecture enables sustained multi-ion release. The glass ionomer phase facilitates the release of fluoride ions (F^−^), while the fluoro-boro-aluminosilicate glass core contributes to the release of additional ions, including strontium (Sr^2+^), borate (BO_3_^3−^), sodium (Na^+^), silicate (SiO_3_^2−^), and aluminum (Al^3+^) (Figure 1B). Although the precise mechanisms governing ion release are not yet fully elucidated, evidence suggests that the presence of the pre-reacted glass ionomer phase surrounding the glass core plays a central role in regulating ion diffusion and sustained release [57].

Currently, a wide range of commercial dental products incorporate S-PRG technology, reflecting its versatility and expanding applications in preventive and restorative dentistry.

One of the first authors to describe the development process that led to the S-PRG particle was Ikemura, R. Tay [58]. In that article, a review of fluoride-releasing adhesives was conducted based on original research articles, review papers, and patent literature. The reports describe technological challenges in developing materials different from conventional glass ionomer cement and improvements in production technology that made it possible to create a revolutionary technology known as “new pre-reacted glass (PRG) ionomer” Roberts, Miyai [59].

Many products developed by Shofu Inc. (Kyoto, Japan) containing the S-PRG particle are commercially available (composite resins, adhesives, resin cements, coating resins, fissure sealants, and polishing pastes), and other products are under development (inorganic cements, root canal sealers, denture bases, tissue conditioners, denture adhesives, toothpastes, varnishes, CAD/CAM composites, and toothbrush filaments). This wide range of applications is based on the ability of the S-PRG particle to release six types of ions (fluoride, strontium, borate, sodium, silicate, and aluminum). Some authors have reported that the concentrations, especially of borate, strontium, and fluoride, are relatively high compared with conventional glass ionomer cement [60].

Another particularly interesting characteristic is that the six released ions do not form salts and are found freely separated in solution. These released ions are responsible for conferring several therapeutic effects to the S-PRG particle, which are useful for restorative treatment [61], caries prevention/management [62], vital pulp therapy [63], endodontic treatment [20], root perforation repair [64], and prevention/treatment of periodontal disease [19].

### 3.3. Dilution and Delivery Vehicle

Determining the non-toxic concentration of any active principle intended for use is one of the initial steps in establishing its biological properties. Accordingly, one of the researchers’ objectives regarding the S-PRG particle was to quantitatively determine ion release from the particle. Fujimoto, Iwasa [65], using inductively coupled plasma atomic emission spectroscopy (ICP-AES), identified the elements released (Al, B, Na, Si, Sr, and F) at different dilutions in distilled water or lactic acid solution. The authors demonstrated that the S-PRG particle released multiple types of ions and that ion release was influenced by the particle-to-solution ratio rather than by the initial pH of the solution.

However, Kashiwagi, Inoue [66] investigated which dilution of the S-PRG particle would be optimal for human gingival fibroblasts (HGFs). Their experiment showed that the undiluted S-PRG eluate exhibited strong cytotoxicity due to high ion concentrations, whereas a 1:100 dilution was considered safe for the tested cells. The dilution medium used was α-modified Eagle’s minimum essential medium (α-MEM), the same culture medium used in the experiment. These findings highlight the importance of controlling the ion concentration and dilution conditions when interpreting the biological effects of S-PRG eluates, particularly when extrapolating experimental observations to potential clinical applications.

In another study, Ishigure, Kawaki [14], using human dental pulp-derived stem cells (hDPSCs), demonstrated that the ionic balance of the eluate differed depending on the dilution and the solvent used (distilled water, DW, or α-MEM), which in turn affected cytotoxicity, cell morphology, cell proliferation, and hDPSC activity.

These findings suggest that dilution of the S-PRG particle is a critical factor influencing its biological activities and was fundamental for the scientific understanding that supported the later development of products for clinical dental applications.

### 3.4. Biological Activities Associated with the S-PRG Particle

**Figure 2:**
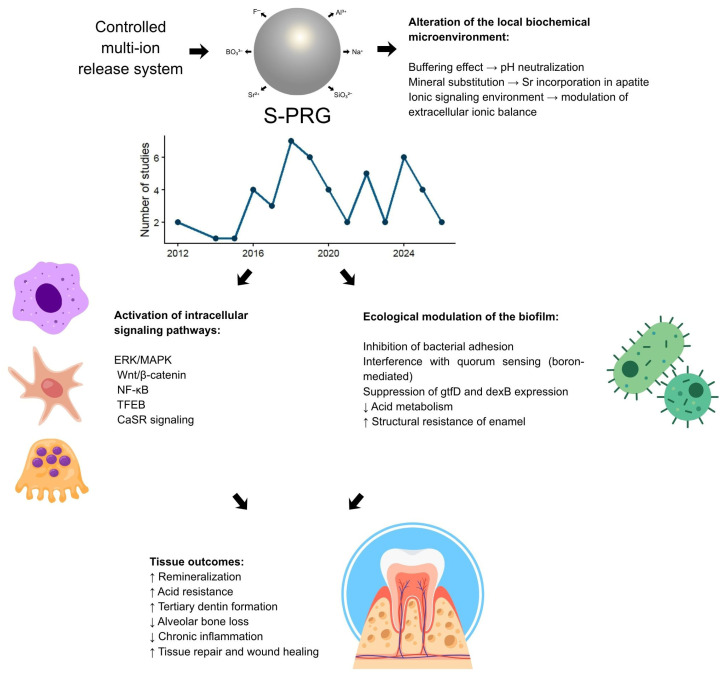
Schematic representation of the hierarchical biological mechanisms associated with surface pre-reacted glass ionomer (S-PRG) particles. The trilaminar structure of the S-PRG filler enables sustained release of multiple ions (F^−^, Sr^2+^, BO_3_^3−^, Na^+^, SiO_3_^2−^, and Al^3+^), which modulate the local chemical microenvironment through buffering effects, mineral substitution, and ionic balance regulation. Based on the studies included in this review, with their temporal distribution between 2012 and 2026, this ion-mediated environment functions as a bioinstructive signaling platform that activates intracellular pathways, including ERK/MAPK, Wnt/β-catenin, NF-κB, and TFEB. These mechanisms promote epithelial barrier integrity, odontogenic and osteogenic differentiation, controlled inflammatory modulation, and the inhibition of osteoclastogenesis. Concomitantly, antimicrobial effects occur through suppression of bacterial adhesion, quorum-sensing interference, and reduced acidogenic metabolism, leading to ecological biofilm modulation. The integration of these chemical, microbial, and cellular responses results in enhanced remineralization, acid resistance, tissue repair, and regenerative potential.

The biological effects associated with the surface pre-reacted glass ionomer (S-PRG) particle are primarily mediated by its multi-ion release system, which includes fluoride (F), strontium (Sr), boron (B), sodium (Na), aluminum (Al), and silicon (Si). These ions interact with biological tissues at chemical, microbial, cellular, and molecular levels, resulting in mineralization, antimicrobial activity, the modulation of inflammation, and the promotion of tissue repair (Figure 2).

#### 3.4.1. Ion-Mediated Mineralization and Enamel Protection

One of the earliest described biological effects of S-PRG particles was their ability to modulate environmental pH through ion release, shifting acidic conditions toward neutral or mildly alkaline values [65]. This buffering capacity contributes directly to the prevention of enamel demineralization.

Beyond pH modulation, S-PRG particles promote mineral deposition through sustained fluoride release enabled by ligand exchange mechanisms within the pre-reacted hydrogel matrix [28,57]. Fluoride enhances remineralization and reduces enamel solubility.

Strontium plays a complementary role by incorporating into the apatite crystal lattice, partially substituting calcium and forming strontium-substituted apatite, which presents increased resistance to acid dissolution [67]. This ion substitution strengthens enamel against cariogenic challenges.

Experimental studies support these mechanisms. Nakamura, Hamba [41] demonstrated that pastes containing 10% S-PRG significantly inhibited enamel demineralization and confirmed strontium incorporation on enamel surfaces. Similar findings were reported by Amaechi, Key [36], Amaechi, Kasundra [42], who showed reduced caries formation in human enamel blocks treated with S-PRG-containing dentifrices. Suge and Matsuo [40] further confirmed that toothpaste formulations with 30 wt% S-PRG effectively inhibited enamel demineralization in vitro. In addition, S-PRG-based cement showed lower demineralization depth, reduced mineral loss, and higher resistance to acidic challenge compared to other cements [51]. S-PRG coatings also significantly increased integrated OCT values and prevented primary enamel demineralization over time [53]. Furthermore, S-PRG pastes exhibited a dose-dependent acid-neutralizing effect, reduced enamel demineralization, and improved hardness, elastic modulus, and surface smoothness [54]. Clinically, Wakamatsu, Ogika [3] observed significant remineralization of white spot lesions in children treated with an S-PRG-containing coating material. However, this study involved a small sample size and lacked a randomized controlled design, which limits the strength of the clinical inference and highlights the need for further well-designed clinical trials to confirm the remineralization potential of S-PRG-based materials.

In addition to enamel protection, S-PRG-based formulations have demonstrated beneficial effects on dentin. Toothpastes containing 5–30% S-PRG fillers reduced dentin hydraulic conductance, indicating decreased dentin permeability Mosquim, Zabeu [45]. Clinical and experimental evidence also shows that S-PRG-containing coatings can significantly reduce dentin hypersensitivity over time [46], which is consistent with their ability to promote complete or partial occlusion of dentinal tubules and maintain this protective effect even after acid exposure [47]. Additionally, S-PRG pastes promoted dentin remineralization, improved mechanical properties, and induced dentinal tubule occlusion after a remineralization period [52].

Beyond these effects, S-PRG eluate also enhanced intrafibrillar remineralization of demineralized dentin, improved collagen ultrastructure, and significantly increased ultimate tensile strength compared to sodium fluoride, indicating structural and biomechanical recovery of the dentin matrix [43].

Most of the evidence supporting these mechanisms derives from in vitro and laboratory-based studies, which provide mechanistic insight but limited direct clinical extrapolation. While these findings consistently demonstrate the biological potential of S-PRG, their translation into predictable clinical outcomes remains dependent on further well-designed clinical investigations. Collectively, these findings demonstrate that S-PRG promotes enamel protection and dentin stabilization through buffering, mineral substitution, sustained ion release, and dentinal tubule occlusion.

#### 3.4.2. Antimicrobial and Antifungal Mechanisms

The antimicrobial activity of S-PRG particles represents another major biological function. Suppression of microbial activity is essential for controlling caries and periodontal disease, and S-PRG technology contributes through multiple mechanisms.

##### Biofilm Disruption and Bacterial Suppression

Several studies demonstrated antibacterial effects against *Streptococcus mutans*, *Enterococcus faecalis*, *Actinomyces israelii*, *Propionibacterium acnes*, *Porphyromonas gingivalis*, and *Fusobacterium nucleatum* [23,27,28,29,35,68,69].

In an in situ human biofilm model, representing an intermediate level of translational relevance between in vitro and clinical conditions, S-PRG-containing prophylaxis paste reduced the *S. mutans*/*S. sanguinis* ratio across different biofilm layers and increased strontium and aluminum incorporation, suggesting ecological modulation and bioactive caries-preventive effects [38].

S-PRG reduces bacterial adhesion, inhibits coaggregation, and suppresses biofilm formation. Boron may interfere with quorum sensing, an essential mechanism for biofilm maturation [35]. Additionally, ion release modulates enzymatic activity and metal ion availability, impairing bacterial metabolism.

Saku, Kotake [68] demonstrated that a resin composite containing S-PRG (Beautifil II, Shofu Inc.) significantly reduced *S. mutans* counts, particularly in the presence of saliva, highlighting the interaction between S-PRG ions and oral fluids in plaque control.

In endodontic applications, S-PRG-containing cements showed antibacterial activity against *E. faecalis* [20], while sustained boron and strontium release was associated with improved infection control and periapical healing [21].

In a human-derived oral microcosm model, daily exposure to S-PRG eluate reduced total microorganisms, mutans streptococci, biofilm accumulation, and lactic acid production, indicating suppression of cariogenic activity in complex biofilms [25].

In a 39-species subgingival biofilm model associated with periodontitis, S-PRG-containing composite resins significantly reduced total bacterial counts, periodontopathogens, and the proportion of Yellow and Orange complexes, indicating modulation of pathogenic biofilm ecology [31].

In addition to inhibiting bacterial growth, PRG Barrier Coat reduced *Streptococcus mutans* adhesion and suppressed the expression of caries-related genes involved in insoluble glucan synthesis (e.g., gtfD and dexB), resulting in altered biofilm structure despite no significant reduction in total bacterial counts [39].

The majority of these antimicrobial findings are derived from in vitro and controlled biofilm models, which allow detailed evaluation of microbial interactions but may not fully represent the complexity of oral ecosystems.

##### Oxidative Stress-Mediated Antifungal Activity

Antifungal effects, particularly against *Candida albicans*, have also been reported [32,33,70,71].

Tamura, Cueno [32] demonstrated that eluates from S-PRG reduced hydrogen peroxide levels and catalase activity in *C. albicans*, inducing oxidative stress. This resulted in suppression of fungal growth, inhibition of biofilm formation, reduced adhesion to denture base resin, inhibition of dimorphic transition, and decreased production of secreted aspartyl proteinases. These findings suggest that S-PRG ions impair fungal virulence and may contribute to the prevention of oral candidiasis, particularly in elderly populations.

#### 3.4.3. Cell Migration, Differentiation, and Regenerative Signaling

Beyond antimicrobial effects, S-PRG particles modulate cellular signaling pathways involved in tissue repair and regeneration.

Nemoto, Chosa [7] reported increased expression of alkaline phosphatase in human mesenchymal stem cells treated with diluted S-PRG eluates, indicating enhanced osteogenic differentiation. Similarly, Okamoto, Ali [16] observed increased expression of CXCL12 and TGFB1 in human dental pulp stem cells, promoting tertiary dentin formation. Comparable differentiation-inducing effects were also described by Kawashima, Hashimoto [72], Miyano, Mikami [73].

The Wnt/β-catenin pathway has also been implicated. Ali, Okamoto [15] showed that the addition of lithium to an S-PRG-containing cement enhanced odontogenic differentiation of human mesenchymal stem cells and promoted reparative dentin formation in vivo through activation of canonical Wnt signaling.

Yamaguchi-Ueda, Akazawa [17] demonstrated that diluted S-PRG eluates (1:10,000) promoted migration of human gingival fibroblasts (HGF-1) via activation of the ERK signaling pathway. This mechanism is associated with wound healing and connective tissue repair.

In a transdentinal model using odontoblast-like cells, S-PRG eluate upregulated dentinogenesis-related genes, including Col1a1, Alpl, Dspp, and Dmp1, and significantly enhanced mineralization without inducing cytotoxic effects [44], which suggests the activation of odontogenic differentiation pathways under clinically relevant conditions.

These findings indicate that S-PRG particles can activate the signaling pathways associated with regenerative processes. However, most of the available evidence derives from in vitro or preclinical models, and the translation of these mechanisms into predictable clinical regenerative outcomes remains to be confirmed.

#### 3.4.4. Modulation of Inflammatory and Matrix Remodeling Processes

Inflammatory regulation is another key biological activity of S-PRG particles. Iwamatsu-Kobayashi, Abe [19] demonstrated reduced collagen destruction and decreased inflammatory cell infiltration in a ligature-induced periodontal disease model treated with S-PRG eluates. Miyaji, Mayumi [20] reported reduced macrophage (CD68) infiltration and antibacterial activity in vivo using S-PRG-containing endodontic cement.

At the molecular level, Thein, Hashimoto [5] observed decreased mRNA expression of pro-inflammatory cytokines (IL-1α, IL-6, TNF-α) in LPS-stimulated macrophages treated with S-PRG dilutions.

Matrix metalloproteinases (MMPs), central regulators of connective tissue remodeling, are also modulated by S-PRG. Inoue, Lan [4], Yamaguchi-Ueda, Akazawa [17] showed that S-PRG modulated secretion of MMP-1 and MMP-3 via ERK activation while reducing TNF-α expression. Moroto, Inoue [6] further demonstrated controlled modulation of MMP-1 production in dental pulp fibroblast-like cells.

Beyond modulation of inflammatory mediators and matrix remodeling, S-PRG eluate also inhibited RANKL-induced osteoclastogenesis in RAW264.7 cells, suppressing NFATc1 expression and MAPK signaling (ERK, JNK, p38), resulting in reduced mineral dissolution. These findings suggest a potential anti-resorptive effect that may contribute to the prevention of alveolar bone loss associated with root caries and periodontal inflammation [18].

Importantly, S-PRG does not simply suppress inflammation; rather, experimental evidence suggests that it may regulate inflammatory and matrix responses in a controlled manner. However, the clinical relevance of these findings remains to be established.

#### 3.4.5. Oral Epithelial Barrier Protection

Gene expression modulation represents an additional biological dimension. Takeuchi, Kato [22] demonstrated that S-PRG-derived ions increased CXADR gene expression via activation of the transcription factor TFEB. The CXADR protein enhances epithelial barrier integrity and limits bacterial virulence factor penetration into subepithelial tissues.

This mechanism suggests a protective role of S-PRG in maintaining epithelial homeostasis, particularly relevant in periodontal disease and mucosal lesions.

### 3.5. Evidence Distribution According to Study Subclass

The methodological classification of the included studies revealed a clear predominance of in vitro investigations, with comparatively fewer in vivo animal studies, in situ human models, and clinical investigations. This distribution reflects the current stage of translational development of S-PRG technology, which remains largely supported by mechanistic and preclinical evidence. Several of the included studies were conducted under controlled experimental conditions, including simplified biofilm models, limited sample sizes, and specific eluate dilutions that may not fully reproduce the complexity of the oral environment. These methodological characteristics should be considered when interpreting the translational applicability of the reported findings.

Most of the included studies were conducted in vitro and focused on cellular responses, biofilm modulation, ion release dynamics, and molecular signaling pathways. These investigations consistently demonstrated that ions released from S-PRG fillers modulate intracellular signaling pathways such as ERK1/2, MAPK, and Wnt/β-catenin, regulate inflammatory mediators and matrix metalloproteinase expression, promote odontogenic and osteogenic differentiation, inhibit bacterial growth and biofilm formation, suppress fungal virulence, and enhance mineral deposition and acid resistance. The principal strength of this subclass lies in its capacity to elucidate biological mechanisms and to isolate the specific contributions of individual ions, including boron, strontium, and fluoride. However, despite the robustness of mechanistic findings, in vitro models inherently lack the complexity of the oral environment, where salivary flow, immune modulation, microbial ecology, and biomechanical factors interact dynamically. Therefore, extrapolation of these findings to predictable clinical efficacy must be approached cautiously.

A smaller number of studies employed in vivo animal models, including rat models of tertiary dentin formation, ligature-induced periodontal disease models, osteoclastogenesis assays, and endodontic inflammatory models. These investigations demonstrated that S-PRG eluates can reduce alveolar bone loss, suppress inflammatory cell infiltration, promote dentin formation, and inhibit osteoclast differentiation. Compared with isolated cell systems, animal studies provide stronger biological validation by incorporating systemic responses and tissue-level interactions. Nevertheless, animal models cannot fully reproduce human oral physiology, long-term restorative performance, or patient-related variables.

In situ human models represent an intermediate translational step between laboratory and clinical research. Studies using enamel slabs or intraoral biofilm devices worn by participants demonstrated modulation of biofilm composition, including reductions in the *S. mutans*/*S. sanguinis* ratio, enhanced fluoride retention, and decreased cariogenic metabolic activity. Because these models operate within the natural oral environment, they provide greater ecological validity than conventional in vitro biofilm systems. However, their relatively short duration and limited sample sizes restrict the strength of long-term clinical inference.

Two clinical investigations were identified evaluating the clinical performance of S-PRG-based materials. In a long-term clinical study, the application of PRG Barrier Coat resulted in a measurable reduction in white spot lesion area after one year of follow-up [3]. Additionally, another clinical investigation evaluating dentin hypersensitivity reported that all tested desensitizing agents reduced hypersensitivity over time, with the S-PRG bioactive varnish showing a significant reduction between 15 and 30 days [46]. Although these findings support the clinical potential of S-PRG-based materials, the limited number of randomized clinical trials indicates that high-level clinical confirmation of their bioactive effects remains insufficient.

Only one clinical investigation with long-term follow-up was identified, demonstrating a measurable reduction in white spot lesion area over one year. Although this finding supports the clinical potential of S-PRG-based materials, the limited number of randomized clinical trials indicates that high-level clinical confirmation of bioactivity remains insufficient.

Overall, the subclass distribution illustrates a typical translational trajectory progressing from mechanistic understanding in vitro to biological validation in vivo, followed by ecological confirmation in situ and limited clinical verification. Although all included studies were classified as Level 2 according to the adopted hierarchy of evidence, their translational strength varies substantially across subclasses. The predominance of in vitro research suggests that S-PRG technology is biologically promising but remains primarily supported by preclinical data. Well-designed randomized clinical trials with long-term follow-up are necessary to determine whether the documented molecular and cellular effects consistently translate into durable and clinically meaningful outcomes.

### 3.6. Comparison of S-PRG Materials with Other Bioactive and Conventional Dental Materials

Some studies have compared S-PRG-containing materials with other conventional or bioactive dental materials, highlighting both similarities and specific advantages associated with S-PRG technology. In a clinical microbiological study, S-PRG composite resins (Beautifil II, LS, and Bulk) showed reduced total bacterial counts and lower levels of periodontopathogens compared with conventional composite resins, suggesting a potential antimicrobial advantage [31]. When compared with sodium fluoride (NaF), the S-PRG filler eluate demonstrated enhanced intrafibrillar mineralization, improved collagen morphology, and increased phosphate/amide ratio and ultimate tensile strength, indicating favorable effects on dentin biomineralization [43]. In dentin permeability studies, toothpastes containing 5–30% S-PRG fillers reduced dentin hydraulic conductance similarly to NaF toothpaste, although NaF varnish initially produced a greater reduction that decreased after erosive challenge [45]. In the context of dentin hypersensitivity management, S-PRG-based materials showed comparable performance to established desensitizers. For example, S-PRG Barrier Coat produced a progressive reduction in dentin hypersensitivity similar to other desensitizing agents such as Duraphat, Biosilicate, and Single Bond Universal Ramos, Briso [46]. Additionally, when compared with Gluma desensitizer, PRG Barrier Coat promoted effective dentinal tubule occlusion and maintained this effect even after acid exposure [47]. Furthermore, when S-PRG-containing resin composites (Beautifil II, Beautifil II Enamel, and Beautifil II LS) were compared with a conventional composite resin (Filtek Z350 XT), the S-PRG materials increased the pH of the surrounding medium over time, indicating a potential buffering effect. Although erosive and abrasive challenges increased surface roughness, S-PRG composites showed improved gloss values [49]. Regarding adhesive performance, S-PRG incorporation did not significantly affect bond strength, except for a reduction observed at higher concentrations (13 wt%) [56], suggesting that its inclusion is generally compatible with adhesive systems within appropriate concentration ranges. Collectively, these findings suggest that S-PRG materials generally show comparable or, in some cases, enhanced biological and functional effects relative to conventional fluoride-based or desensitizing materials.

## 4. Conclusions

Based on the evidence gathered in this integrative review, the S-PRG (Surface-reaction-type prereacted Glass ionomer) particle demonstrates consistent bioactive properties mediated by its sustained multi-ion release system. These biological effects extend across multiple domains, including mineralization, antimicrobial activity, inflammatory modulation, epithelial protection, and activation of specific intracellular signaling pathways involved in tissue repair and regeneration.

Although this review consolidates current findings regarding S-PRG particles, important limitations remain. The available literature is still predominantly composed of in vitro and experimental studies, and further well-designed in vivo and preclinical proof-of-concept investigations are required to clarify mechanisms, confirm biological relevance, and establish clinical efficacy.

Notably, processes such as cell migration, differentiation, and gene expression modulation have been consistently associated with ions released from the S-PRG particle. These mechanisms suggest that S-PRG technology holds promising potential for the development of novel bioactive materials with tissue-regenerative properties, supporting both preventive and therapeutic strategies in oral healthcare.

## Figures and Tables

**Table 1:** Summary of the studies included in this integrative review, categorized according to methodological subclass (in vitro, in vivo, in situ human model, and clinical investigation), main biological activity investigated, experimental model, material or vehicle used, key findings, and corresponding references. Although grouped within the same hierarchical level, study subclasses are presented separately to reflect differences in evidentiary strength and translational relevance, with clinical and in situ studies providing higher applicability compared to in vitro and animal models.

Main Activity	Experimental Model	Sample Size	Material/Vehicle	S-PRG Material Dilution	Key Findings	Reference	Study Subclass
Cell proliferation/cytocompatibility	hDPSCs	3–6 wells per group	S-PRG eluate (DW/α-MEM)	1:500, 1:100, 1:10, 1:2	Cell responses were dilution-dependent; modulation of proliferation and ALP activity	Ishigure, Kawaki [14]	In vitro
Tertiary dentin formation (Wnt/β-catenin)	hDPSCs + Wistar rats	Cells: *n* = 3 Rats: 24 total	S-PRG + LiCl cement	1.5:1.0 wt (S-PRG Powder/FGL liquid)	Increased migration, differentiation, mineralization; induced tertiary dentin via Wnt/β-catenin signaling	Ali, Okamoto [15]	In vivo
Tertiary dentin formation	hDPSCs + rats	Cells: *n* = 8 Rats: *n* = 3	S-PRG cement	1.5:1.0 wt (S-PRG Powder/liquid)	Induced dentin formation comparable to MTA; regulated osteo/dentinogenic genes	Okamoto, Ali [16]	In vivo
Osteogenic differentiation	hMSCs	*n* = 3, 6, or 8	S-PRG eluate	1:2–1:1000	Upregulated ALP and mineralization (dose-dependent, no cytotoxicity)	Nemoto, Chosa [7]	In vitro
Tissue remodeling	Human pulp fibroblasts	*n* = 4	S-PRG eluate ± MDP	1:1, 0.01%, 0.1%, and 1%	Enhanced MMP-1 production via ERK; CaSR-dependent modulation	Moroto, Inoue [6]	In vitro
Wound healing/cell migration	HGF-1 cells	*n* = 4, 8, or 10	S-PRG eluate (1:10,000)	1:100, 1:1000 and 1:10,000	Promoted migration via ERK1/2 activation	Yamaguchi-Ueda, Akazawa [17]	In vitro
Anti-osteoclastogenic/bone resorption inhibition	RAW264.7 cells (RANKL-induced osteoclastogenesis)	Not clear	S-PRG eluate (1:200–1:400)	1:10–1:1200	Suppressed OC formation and mineral dissolution; downregulated NFATc1, OCSTAMP, CATK; inhibited ERK/JNK/p38 signaling	Chandra, Nakamura [18]	In vitro
Inflammatory modulation	Human gingival fibroblasts	*n* = 4	S-PRG eluate	1:1, 0.01–1%	Regulated MMP-1/MMP-3 secretion and ERK/p38 signaling	Inoue, Lan [4]	In vitro
Anti-inflammatory (macrophages)	RAW264.7 cells	*n* = 3 or 4	S-PRG sealer extract	1:2, 1:4	Downregulated IL-1α, IL-6, TNF-α and p-NF-κB	Thein, Hashimoto [5]	In vitro
Anti-inflammatory/periodontal protection (in vivo)	Mouse ligature-induced periodontal disease model	*n* = 3	S-PRG eluate	10 μL S-PRG eluate	Reduced alveolar bone loss; decreased neutrophil and macrophage infiltration; preserved collagen bundles; boron ion deposition detected	Iwamatsu-Kobayashi, Abe [19]	In vivo
Antibacterial + anti-inflammatory	Cells + Wistar rats	Cells: *n* = 5 Rats = 11 total	S-PRG sealer	-	Reduced *E. faecalis* growth and in vivo inflammatory response	Miyaji, Mayumi [20]	In vivo
Ion release/osteogenic potential	Extracted human teeth	*n* = 6	Prototype S-PRG sealer	-	Released boron and strontium ions; potential antimicrobial and osteogenic effects	Bhat, Cvach [21]	In vitro
Epithelial barrier protection	3D gingival epithelium	*n* = 2	S-PRG eluate	1:1, 0–25 μL per well	Upregulated CXADR via TFEB; reduced bacterial permeation	Takeuchi, Kato [22]	In vitro
Antibacterial (*S. mutans*)	*S. mutans*	*n* = 5	Resin composite with S-PRG	1:1, 50, 25, and 12.5 (vol.%)	Growth inhibition is concentration-dependent; BO_3_^3−^ and F^−^ most active ions	Miki, Kitagawa [23]	In vitro
Antibacterial/anti-biofilm	Multispecies biofilm	*n* = 3	SPRG-filled RBC + MPC	MPC (1.5–10% by weight).	Reduced protein adsorption and biofilm formation; improved acid neutralization	Lee, Kwon [24]	In vitro
Antibacterial/anti-biofilm (microcosm)	Human-derived oral microcosm biofilm (enamel specimens, 120 h)		S-PRG eluate	100%	Reduced total microorganisms, streptococci and mutans streptococci; decreased lactic acid production; reduced biofilm structure (SEM)	Garcia, Namba [25]	In situ human model
Antifungal	*Candida spp.* + *G. mellonella*	*n* = 3	S-PRG eluate	1:1, 50, 40, 30, 20, 10, and 5%,	Reduced biofilm and virulence; protective in vivo effect	Rossoni, de Barros [26]	In vivo
Oral biofilm modulation	Human saliva biofilm	*n* = 4	S-PRG eluate	1:1, 10, 20, 30, 40, 50, 60, 70, 80, 90, and 100%	Suppressed biofilm formation and VSC production	Suzuki, Yoneda [27]	In situ human model
Antimicrobial/coaggregation	Oral bacteria	*n* = 3	S-PRG eluate	1:1, 20% and 50%	Reduced bacterial growth and coaggregation	Shimazu, Oguchi [28]	In vitro
Antibacterial (gene expression)	*S. mutans*	*n* = 4	S-PRG eluate	1:1, 25%, 12.5% and 6.3%	Downregulated sugar metabolism operons; reduced cariogenicity	Nomura, Morita [29]	In vitro
Antibacterial toothbrush filament	*S. mutans*	Not clear	S-PRG-containing monofilament	Monofilaments containing S-PRG filler	Reduced biofilm; stronger effect in nylon	Matayoshi, Nomura [30]	In vitro
Antibacterial and anti-biofilm activity/coaggregation modulation	Oral bacteria (*S. gordonii*, *S. mutans*, *S. oralis*, *L. acidophilus*, *C. albicans*)	*n* = 3	S-PRG eluate	1:1, 0–50%	Reduced bacterial growth and biofilm formation; inhibited coaggregation of *S. gordonii* with *S. oralis and F. nucleatum*; increased autoaggregation of *S. gordonii* at specific concentrations	Shimazu, Oguchi [28]	In vitro
Antibacterial/subgingival multispecies biofilm	39-species periodontitis-associated biofilm (7 days, 96-well plate)	*n* = 12	S-PRG composite resins (Beautifil II, LS, Bulk) vs. conventional composite	-	Reduced total bacterial counts; decreased periodontopathogens and Yellow/Orange complexes; lower metabolic activity	de Lima, de Cassia Orlando Sardi [31]	In vitro
Antifungal (oxidative stress)	*C. albicans*	Not clear	S-PRG ion extraction liquid (ELIS)	1:1, 1:8, 1:16, 1:32, 1:128	Induced oxidative stress; reduced growth, biofilm and virulence factors	Tamura, Cueno [32]	In vitro
Antifungal/denture relining material	Hard denture liner with S-PRG (micro/nanofillers)	*n* = 4, 5 or 20	S-PRG filler	2.5–20 wt%	10 wt% nanofiller reduced C. albicans adhesion while maintaining mechanical properties	Sunami, Inokoshi [33]	In vitro
Fluoride retention in biofilm	In situ oral biofilm	Not clear	S-PRG toothpaste	1:3	Enhanced fluoride retention via mineral ion uptake	Kato, Tamura [34]	In situ human model
Anti-caries (bacterial adherence)	*S. mutans*/*P. gingivalis*	*n* = 3	S-PRG eluate	10, 50 and 100%	Reduced adherence and proteolytic activity	Yoneda, Suzuki [35]	In vitro
Anti-caries (demineralization)	Human enamel blocks	*n* = 30	S-PRG toothpaste	0–20%	Reduced enamel demineralization	Amaechi, Key [36]	In vitro
Anti-caries varnish	Bovine enamel	*n* = 15	S-PRG varnish	10–40%	Dose-dependent protection against demineralization	Spinola, Moecke [37]	In vitro
Anti-caries/biofilm modulation (in situ)	In situ enamel slab device (human participants, 5 days)	*n* = 9 or 10	S-PRG-containing prophylaxis paste filtrate	1:3	Reduced *S. mutans/S. sanguinis* ratio across biofilm layers; increased strontium and aluminum incorporation	Kato, Kutsuna [38]	In situ human model
Anti-caries/biofilm gene modulation	*S. mutans* biofilm on hydroxyapatite disks	*n* = 3	PRG barrier coat	-	Reduced adhesion; suppressed caries-related gene expression (gtfD, dexB); altered biofilm structure	Nishimata, Kamasaki [39]	In vitro
Acid resistance/crystallinity	Hydroxyapatite pellets	Not clear	S-PRG toothpaste	0–30 wt%	Increased crystallinity and acid resistance	Suge and Matsuo [40]	In vitro
Demineralization inhibition	Bovine enamel	Not clear	S-PRG paste	0–30 wt%	S10 showed the greatest protective effect	Nakamura, Hamba [41]	In vitro
pH-cycling caries model	Tooth blocks	n = 7	S-PRG toothpaste	1–30%	Up to ~70% reduction in demineralization	Amaechi, Kasundra [42]	In vitro
Clinical remineralization (WSLs)	Children’s teeth	7 children, 17 teeth	PRG Barrier Coat	-	Reduced white spot lesion area over 1 year	Wakamatsu, Ogika [3]	Clinical investigation
Dentin remineralization/collagen reinforcement	Demineralized bovine dentin (3-month SBF storage)	*n* = 8	S-PRG filler eluate vs. NaF	1:1	Enhanced intrafibrillar mineralization; improved collagen morphology; increased phosphate/amide ratio and UTS	Ubolsa-ard, Sanon [43]	In vitro
Transdental odontogenic stimulation	MDPC-23 cells + artificial pulp chamber (dentin disk model)	*n* = 2, 8 or 10	S-PRG filler eluate	20 μL	Promoted odontogenic gene expression and enhanced mineralization (~40%) after prolonged exposure	Mendes Soares, Anselmi [44]	In vitro
Dentin permeability/hydraulic conductance	Human dentin disks from third molars	*n* = 8	Toothpastes containing S-PRG fillers vs. NaF toothpaste and NaF varnish	0–30%	Toothpastes containing 5–30% S-PRG reduced dentin hydraulic conductance similarly to NaF toothpaste; NaF varnish showed greater initial reduction but the effect decreased after erosive challenge	Mosquim, Zabeu [45]	In vitro
Dentin hypersensitivity reduction	Human teeth with non-cavitated root exposure (clinical evaluation using VAS and CoVAS)	Np-11, Nd = 48	S-PRG barrier Coat vs. Duraphat, Biosilicate, and Single Bond Universal.	-	All desensitizers reduced dentin hypersensitivity over time; the S-PRG bioactive varnish showed a significant reduction between 15 and 30 days	Ramos, Briso [46]	Clinical investigation
Dentinal tubule occlusion/dentin hypersensitivity prevention	Human dentin disks evaluated by SEM and EDS after acid challenge	*n* = 10	PRG Barrier Coat (S-PRG filler) vs. Gluma desensitizer and controls	-	PRG Barrier Coat promoted complete or partial dentinal tubule occlusion and maintained the effect after acid exposure	Ribeiro, Ferreira [47]	In vitro
Ion release and physicomechanical properties of denture base resin	PMMA resin specimens (disk and rectangular)	72 disk specimens and 32 rectangular specimens	PMMA resin containing S-PRG nanoparticles	5 wt% and 10 wt% S-PRG nanoparticles; 20 wt% S-PRG microparticles	Incorporation of S-PRG nanoparticles enabled the release of B, Si, Sr, Na, and F ions and maintained flexural strength within standards; 10 wt% showed the best balance between ion release and physicomechanical properties	Ratanakupt, Nakatsuka [48]	In vitro
pH buffering capacity and surface properties of S-PRG resin composites	Resin composite disks exposed to erosive/abrasive cycles	*n* = 5 for pH analysis *n* = 10 for erosion/abrasion test	Resin composites: Filtek Z350 XT (control), Beautifil II, Beautifil II Enamel, Beautifil II LS (S-PRG fillers)	-	S-PRG-containing composites increased the pH of the surrounding medium over time; erosive/abrasive challenge increased surface roughness but gloss values improved in S-PRG composites	Oliveira Neto, Picolo [49]	In vitro
Enamel remineralization of subsurface lesions	Bovine enamel specimens with artificial subsurface lesions	*n* = 50	Gum-base material extracts containing S-PRG filler (GE0, GE5, GE10)	0, 5, 10 wt%	5% and 10% S-PRG groups showed significantly reduced lesion depth and enhanced remineralization; ion deposition confirmed by SEM/EDS	ThanNaing, Hiraishi [50]	In vitro
Anti-demineralization and dentin remineralization	Bovine dentin (crown cavities and root dentin blocks) under pH cycling	*n* = 32 teeth (crown dentin) + 64 root dentin blocks	Self-adhesive resin cements: S-PRG-based cement, Si-based cement, and RelyX cement	-	S-PRG-based cement showed lower demineralization depth, reduced mineral loss, and higher resistance to acidic challenge compared to other cements	ThanNaing, Abdou [51]	In vitro
Dentin remineralization and mechanical recovery	Demineralized human dentin blocks	*n* = 15	Pastes containing S-PRG filler vs. nano-hydroxyapatite paste	0, 5, 30%	S-PRG pastes promoted dentin remineralization, improved mechanical properties, and induced tubule occlusion after the remineralization period	Iijima, Ishikawa [52]	In vitro
Enamel anti-demineralization (protective coating)	Extracted human primary teeth evaluated by optical coherence tomography (OCT)	*n* = 18 (3 groups of 6)	Coating material containing S-PRG filler	-	S-PRG coating significantly increased integrated OCT values and prevented primary enamel demineralization over time	Murayama, Nagura [53]	In vitro
Enamel anti-demineralization and acid neutralization	Human enamel blocks exposed to a demineralizing solution	*n* = 38	Pastes containing S-PRG filler vs. non-fluoride and hydroxyapatite pastes	0, 5, 30%	S-PRG pastes showed dose-dependent acid-neutralizing effect, reduced enamel demineralization, and improved hardness, elastic modulus, and surface smoothness	Iijima, Kawaguchi [54]	In vitro
Microtensile bond strength of S-PRG adhesives	Flattened dentin surfaces of extracted human molars	*n* = 25 (5 groups of 5)	Experimental all-in-one adhesives containing S-PRG filler vs. Fluorobond Shakeone (control)	0, 13, 27, 40 wt%	S-PRG incorporation did not significantly affect bond strength, except for a reduction at 13 wt%	Kawashima, Shinkai [55]	In vitro
Pulp healing and tertiary dentin formation	Exposed rat pulp (direct pulp capping, 14 and 28 days)	*n* = 6	Experimental all-in-one adhesives containing S-PRG filler vs. control adhesive	13 and 27 wt%	S-PRG adhesives showed no pulpal inflammation and promoted tertiary dentin formation; 13% and 27% formed dentin bridge comparable to the control after 28 days	Kawashima, Shinkai [56]	In vivo

## Data Availability

No new data were created or analyzed in this study. Data sharing is not applicable to this article.

## Abbreviations

The following abbreviations are used in this manuscript:

hDPSCs	Human dental pulp-derived stem cells
HGF	Human gingival fibroblasts
ICP-AES	Inductively coupled plasma atomic emission spectroscopy
S-PRG	Surface pre-reacted glass ionomer
α-MEM	α-modified Eagle’s minimum essential medium
